# The Unique Sympatric Population of Uzzell’s Lizard (*Darevskia uzzelli*, Lacertidae, Squamata) Reveals Clonal Diversity and Urgent Conservation Value

**DOI:** 10.3390/ani16142140

**Published:** 2026-07-09

**Authors:** Marine Arakelyan, Irena Martirosyan, Oleg Nikolaev, Eugene Iryshkov, Igor Doronin, Çetin Ilgaz, Yusuf Kumlutaş, Angin Grigoryan, Eduard Galoyan

**Affiliations:** 1 Research Institute of Biology, Yerevan State University, Yerevan 0025, Armenia; 2 The Laboratory of Genome Organization, Institute of Gene Biology, Russian Academy of Sciences, 119334 Moscow, Russia; irena-m@yandex.ru; 3 The Laboratory of Comparative Ethology and Biocommunication, A.N. Severtsov Institute of Ecology and Evolution, 119071 Moscow, Russia; nikolaevod2002@gmail.com (O.N.); eiryshkov@gmail.com (E.I.); saxicola@mail.ru (E.G.); 4 Faculty of Biology, M.V. Lomonosov Moscow State University, 119234 Moscow, Russia; 5 Laboratory of Herpetology, Zoological Institute of the Russian Academy of Sciences, 199034 Saint Petersburg, Russia; ivdoronin@mail.ru; 6 Department of Biology, Faculty of Science, Dokuz Eylül University, İzmir 35390, Türkiye; cetin.ilgaz@deu.edu.tr (Ç.I.); yusuf.kumlutas@deu.edu.tr (Y.K.); 7 Fauna and Flora Research and Application Center, Dokuz Eylül University, İzmir 35390, Türkiye; 8 Faculty of Mathematics and Natural Science, Shirak State University, Gyumri 3126, Armenia; angingrigoryan70@gmail.com

**Keywords:** Caucasian rock lizards, parthenogenesis, sympatric assemblage, morphology, microsatellites

## Abstract

The identification of the parthenogenetic Uzzell’s lizard, *Darevskia uzzelli* (Darevsky & Danielyan, 1977), in Armenia represents the first record of this species in the country. Before this discovery, *D. uzzelli* was considered an endangered species endemic to a small region of northeastern Türkiye. Genetic and morphological analyses have confirmed that the Armenian lizards are indeed *D. uzzelli*, consistent with specimens from Türkiye. Morphological and genetic data also firmly discriminate *D. uzzelli* from the closely related parthenogenetic species *D. unisexualis*. Our results reveal significant morphological variation in this parthenogen among populations. Notably, the sympatric area in Armenia where *D. uzzelli* has been identified is unique for hosting the richest known assemblage of parthenogenetic lizards. Here, *D. uzzelli* coexists with three other parthenogenetic species already known to inhabit the region: *D. unisexualis*, *D. armeniaca*, and *D. dahli*. This exceptional population requires continuous study and protection due to its high scientific value.

## Simple Summary

Uzzell’s lizard, *Darevskia uzzelli* (Darevsky & Danielyan, 1977), is an endangered parthenogenetic, all-female species and has been recognized as endemic to eastern Türkiye since its first description in 1977. In the present paper, we report the discovery of a Uzzell’s lizard population in Armenia, supported by morphological and genetic analysis. The newly discovered population is the only known one where four unisexual species coexist, and no sexual species were observed. The morphological and genetic analyses revealed significant diversity both within and between populations of Uzzell’s lizard. The discovery of this species in Armenia calls for the protection of this rare lizard population and highlights the global importance of its unique sympatric habitat.

## 1. Introduction

The Caucasian rock lizards of the genus *Darevskia* Arribas, 1999, are widely distributed across the Caucasus ecoregion and include at least six recognized parthenogenetic species together with approximately 32–35 sexually reproductive species, most of which are distributed in the Armenian Highlands [1,2,3]. Among the parthenogenetic species of this genus, *Darevskia sapphirina* (Schmidtler, Eiselt & Darevsky, 1994) and *D. bendimahiensis* (Schmidtler, Eiselt & Darevsky, 1994), together with *D. uzzelli* (Darevsky & Danielyan, 1977), are endemic to eastern Türkiye since their original descriptions [4,5]. The type locality of *D. uzzelli* is situated approximately 25 km south of Kars in northeastern Türkiye. These are small lizards well adapted to climbing on rocks, with a maximum snout–vent length (SVL) of about 65 mm [6]. Due to its restricted distribution and ongoing habitat decline, *D. uzzelli* is currently listed as Endangered on the IUCN Red List of Threatened Species [7].

*Darevskia uzzelli* is a hybrid parthenogenetic species [8,9] that originated during the Pleistocene [1,10]. Genetic studies indicate that *D. uzzelli* and *D. unisexualis* (Darevsky, 1966) are the closest relatives, sharing a maternal lineage from *D. raddei nairensis* (Darevsky, 1967) [9,10]. Two other rare parthenogenetic species from Türkiye, *D. sapphirina* and *D. bendimahiensis*, likely represent a single taxon and are thought to have originated from another maternal subspecies, *D. raddei vanensis*, similar to *D. uzzelli* [10,11].

The known range of *D. uzzelli* is restricted to northeastern Türkiye [12]. Its habitats consist of rocky steppes and river gorges that are geographically continuous from Türkiye into western Armenia, separated by the Akhuryan River. Therefore, Darevsky and Danielyan [4] hypothesized that these lizards could have crossed this riverine boundary and may also occur in Armenia. During fieldwork conducted between 2020 and 2025 in the Akhuryan River valley near the village of Jradzor in Armenia, our team identified individuals coexisting with the parthenogenetic species *D. armeniaca*, *D. unisexualis*, and *D. dahli*. Preliminary morphological and microsatellite genotyping of these individuals suggested that they belong to *D. uzzelli* [13]. The present study aims to verify the taxonomic status of these individuals and to analyze their genetic and morphological variation in comparison with Turkish populations and reference paratype samples of *D. uzzelli*.

## 2. Materials and Methods

**Figure 1:**
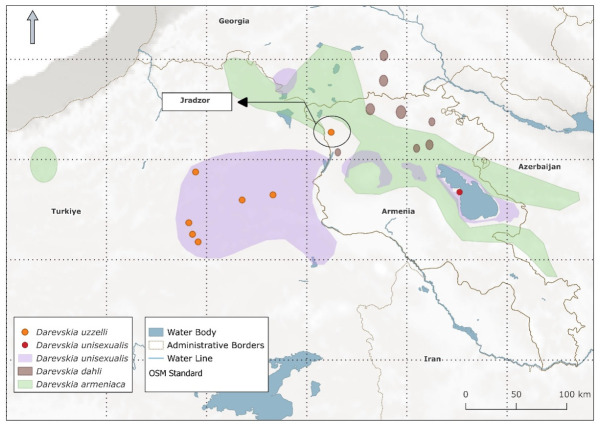
Known localities of *D. uzzelli* (orange circles) and distribution ranges of three parthenogenetic *Darevskia* species according to the currently recognized distribution ranges presented in the IUCN Red List Threatened Species maps (The IUCN Red List of Threatened Species. Version 2025-2. https://www.iucnredlist.org). The black-circled locality of *D. uzzelli* marks the newly recorded sympatric population near Jradzor village in Armenia. The red circle indicates the Noratuz population of *D. unisexualis* used for comparison. The distribution range of *D. unisexualis* is shown in purple, the distribution range of *D. dahli* is shown by brown circles, and the distribution range of *D. armeniaca* is shown in green. Although all four species were recorded together at the Jradzor locality, this area is not included within the currently recognized distribution ranges presented in the IUCN Red List Threatened Species maps.

Two adult female rock lizards with SVLs = 64 and 61 mm, putatively identified as *D. uzzelli*, were captured on 28 June 2020 and 25 May 2025, in the Shirak province of western Armenia, near Jradzor village in the Akhuryan River valley (N40.915131, E43.763127, WGS84) by M.S. Arakelyan and E.A. Galoyan (Figure 1). The female captured in 2025 was temporarily maintained under captive-breeding conditions at the Severtsov Institute of Ecology and Evolution and produced a clutch. The specimens were preserved in 70% ethanol and deposited in institutional collections of the Faculty of Biology, Yerevan State University (YSU 200623-15), and Zoological Museum of Moscow State University (ZMMU Re-18310), respectively.

At the mentioned locality, three other parthenogenetic rock lizard species were morphologically and genetically identified, namely *D. armeniaca*, *D. dahli*, and *D. unisexualis* [13]. The studied specimens were compared morphologically with specimens of *D. uzzelli* from Türkiye and with the closest parthenogenetic relative of this species, *D. unisexualis* from Armenia. Reference specimens included seven *D. uzzelli* paratypes from the Zoological Institute of the Russian Academy of Sciences, Saint Petersburg (ZISP-10815, 1–4, Zanzak, Erzurum, 27 July 1910, P.V. Nesterov; ZISP-18819, 25 km south of Kars, 26 May 1967, R.J. Clark & E.D. Clark; ZISP-21212, 1–2, 15 km southeast of Horasan, 1996, I.S. Darevsky), 12 *D. uzzelli* from Türkiye (N133/2002, YSU-310, 1–12, 28 km southeast of Horasan, Erzurum, 3 September 2002, Çetin Ilgaz and Yusuf Kumlutaş), and 39 specimens of *D. unisexualis* (YSU-230, 1–39, Noratuz village, Lake Sevan, Armenia, 6 June 2010, M.S. Arakelyan) (Figure 1).

A total of 18 morphological measurements and scalation characters were examined following [14,15]. Here, we present only the key characters included in the Section 3: MBSN–number of dorsal scales counted along a transversal line at mid-body; VSN–number of ventral scales along a longitudinal line from the first row that clearly shows four sub-rectangular ventral scales up to the collar; CSN–collar scale number; GSN–gular scale number from the angle between the maxillar scales to the collar; FPN–femoral pore number; SDLN–number of subdigital lamellae from the 4th toe; SCSN–number of supraciliar scales; SCGN–number of supraciliary granules; SMN–number of scales between the masseteric shield and the supratemporal scale; MTN–number of scales between masseteric and tympanum shields; SLN–number of of supralabial scales preceeding the eye; PA–preanal scale number; PTMN–postemporal scale number; aNDSN–average number of dorsal scales along one abdominal scale near the limb end (10 abdominal scales were counted); TibN–number of scales lying on the dorsal surface of the ankle between the large scales on the right hindlimb; FemN–longitudinal rows of scales on the ventral surface of the thigh between the femoral pores and the outer row of enlarged scales on the right hindlimb; Rost + Frns–rostral separated from frontonasal scale (1 yes/2 no). All counts and measurements were taken on the right side of each lizard. Additionally, SVL (length of body from tip of snout to cloaca) with an accuracy of 0.5 mm with a caliper ruler (MITUTOYO, Kanagawa, Japan) was measured for each lizard.

Statistical analysis of scalation features and plotting were performed with R (version 4.4.2) [16] in RStudio (v.2024.12.0 + 467). We applied the FactoMineR package (v.2.11) [17] for Factor Analysis of Mixed Data (FAMD) to analyze morphological variability of the scalation features. This method allows the use of numerical and categorical variables simultaneously and was successfully applied in [18]. Euclidean distances between the centroid of the samples from Jradzor and other studied populations within the FAMD coordinates were calculated using tidyverse (v.2.0.0) [19] and cluster (v.2.1.8.1) [20] packages. Canonical variate analysis was performed using the MASS package (v.7.3-64) [21]. All data were visualized using ggplot2 (v.3.5.2) [22] and factoextra (v.1.0.7) [23] packages.

DNA was extracted from the tail tips of two *D. uzzelli* specimens from Jradzor, preserved in 96% ethanol, and from two museum specimens (ZISP-21212) using the Biolabmix D-Cells kit (BIOLABMIX LLC, Novosibirsk, Russia) and the phenol–chloroform method with proteinase K digestion. Three tetranucleotide microsatellite loci (Du323, Du215, Du47G), previously developed for rock lizards [13,24,25], were studied. Parthenogenetic lizards of the genus *Darevskia* are characterized by species-specific amplification profiles at these loci, as well as species-specific multilocus genotypes formed by their combinations [13]. PCR fragments of each locus were extracted from the gel and sequenced by the Sanger method by Evrogen. The amplified fragment sizes were 189 and 223 bp for locus Du323, 192 and 227 bp for Du215, and 148, 168, and 172 bp for Du47G. Alleles were confirmed by bidirectional Sanger sequencing using forward and reverse primers.

In addition, four mitochondrial loci were amplified: cytochrome b (*cyt b*; primers L14724 and H15175) [26], cytochrome oxidase I (COI; primers LCO1490 and HCO2198) [27], NADH dehydrogenase subunit 4 (ND4; primers ND4 and Gram_A) [28], and 12S rRNA (primers 12Sal and 12Sbh) [27]. These markers were selected due to the availability of comparative sequence data for *Darevskia* species in GenBank for reliable phylogenetic and population-level comparison. Microsatellite loci were analyzed based on fragment length variation and sequence polymorphisms. Multilocus genotypes were reconstructed from allele combinations across loci, representing clonal lineages characteristic of parthenogenetic reproduction.

Sequences were read, assembled, and aligned in UGENE 49.1 [29], and subsequently analyzed using NCBI BLAST (BLAST+ 2.17.0: July 22, 2025) for species identification and similarity assessment. The resulting sequences were deposited in NCBI GenBank under accession numbers PX514661–PX514666 and PX845416 (microsatellite alleles) and PX514667–PX514670 (mitochondrial loci) and compared with previously published sequences of *D. uzzelli* from Kars (ZISP-21212-2; GenBank: MN613706, MN613799, MN613613) and Erzurum (GenBank: MN211062). The nucleotide sequences of all detected alleles at loci Du215, Du323, and Du47G were compared between *D. uzzelli* and *D. unisexualis* sequences available in NCBI (GenBank Acc. KX258628–KX258630, KX258637, KX258638, KX258639–KX258641).

## 3. Results

**Figure 2:**
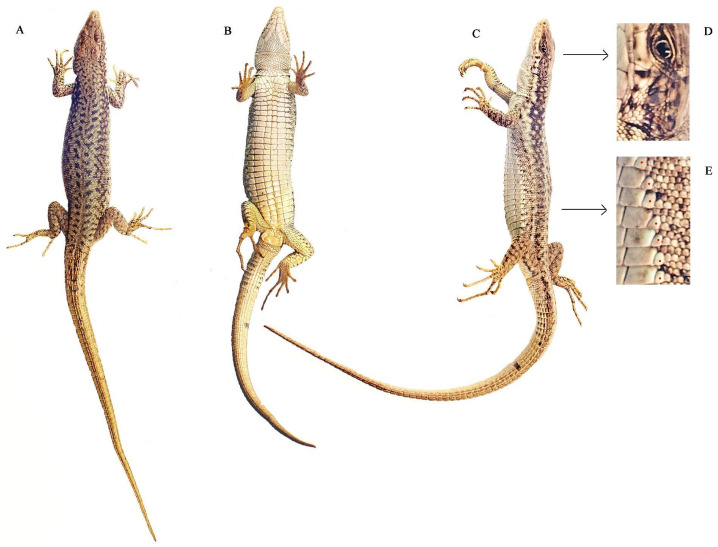
Live views of (**A**) the dorsal, (**B**) belly, and (**C**) lateral body sides of *D. uzzelli* ZMMU Re-18310 from the Jradzor population in Armenia. (**D**) Lateral view of the head, where enlarged scales between masseteric and tympanical shields are shown with red dots, (**E**) ventral part of the body with enlarged dorsal scales near the border with ventral scales (red dots). The snout–vent length of the specimen is 61 mm.

The results of the analysis of scalation and color patterns in two lizards collected in Armenia showed that they are consistent with the characteristics of the paratypes of *D. uzzelli* [4,5]. Dorsally, the specimens exhibited a greenish-brown coloration with a prominent, broad dark pattern consisting of distinct blotches along the vertebral line and two dark stripes extending from the tympanum area caudally along the flanks. Ventrally, the belly and throat were white, while the femoral and cloacal regions showed a yellowish color (Figure 2).

The Armenian two lizards show pholidosis similar to other populations of *D. uzzelli* (Table 1). They are characterized by smooth dorsal scales, the absence of contact between the rostral and frontonasal scales, the presence of enlarged scales between the masseteric and tympanic scales, and two large, approximately equal-sized posttemporal shields. Between the supraocular and supraciliary shields on each side, there are respectively 12 and 14 granules, forming a continuous row. The collar is well developed, smooth-edged, and composed of 12 scales. The ventral scales laterally contact three body scales, of which the posterior one is distinctly enlarged and often triangular in shape. The anal plate is preceded by two enlarged preanal scales. Additionally, all *D. uzzelli* lacked mating scars on the belly, which typically indicate an absence of recent mating activity [14]. One captured female laid a clutch of two eggs on 4 July 2025.

**Figure 3:**
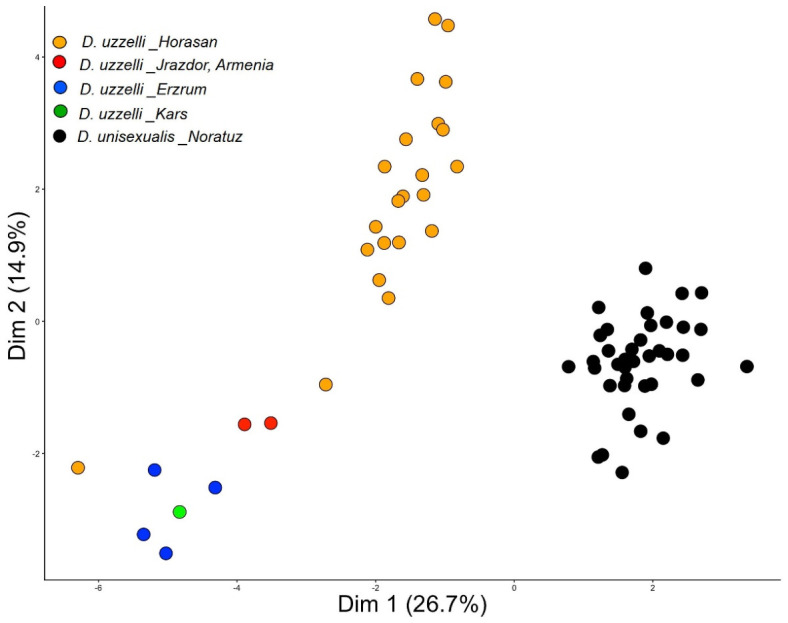
FAMD dimension plot of the scalation: *D. uzzelli* from Jradzor, Armenia (N = 2) is indicated in red; Kars, Türkiye (N = 1)—green, Horasan, Türkiye (N = 22)—orange, and Erzurum, Türkiye (N = 4)—blue. For comparison, *D. unisexualis* from Noratuz, Armenia (N = 39) is shown in black.

The features of pholidosis of *D. uzzelli* showed considerable variation both among and within populations (Table 1, Figure 3). Factor Analysis of Mixed Data (FAMD) indicated that the first two dimensions explained 26.7% and 14.9% of the morphological variation among the studied groups, respectively. The Armenian population was morphologically closer to the Kars and Erzurum populations than to the Horasan population (Euclidean distances from the centroid: 1.75, 1.84, and 3.96, respectively) and was clearly more distant from *D. unisexualis* (Euclidean distance from the centroid: 5.59).

Canonical Variate Analysis (CVA) also confirmed a clear separation between *D. unisexualis* and *D. uzzelli* (Shapiro–Wilk test, F = 912.3, *p* < 0.001). In addition, CVA detected significant morphological differences between the Erzurum and Horasan populations of *D. uzzelli* (Shapiro–Wilk test, F = 45.209, *p* < 0.001). Only two individuals from the Horasan population were confused with individuals from Erzurum. Species discrimination based on CVA was 100% accurate.

Mitochondrial sequences (GenBank: PX514667–PX514670) from the Jradzor specimens (ZMMU Re-18310 and YSU 200623-1) matched those of *D. uzzelli* from Kars (ZISP-21212-2; GenBank: MN613706, MN613799, MN613613) [27] and were consistent with sequences reported from Erzurum (GenBank: MN211062) [10]. No private or novel mitochondrial haplotypes were detected in the Jradzor population for the *cyt b*, COI, and 12S loci. For the ND4 locus, a single nucleotide substitution was detected within the 710 bp analyzed fragment; however, this variation was insufficient to define a distinct haplotype.

**Figure 4:**
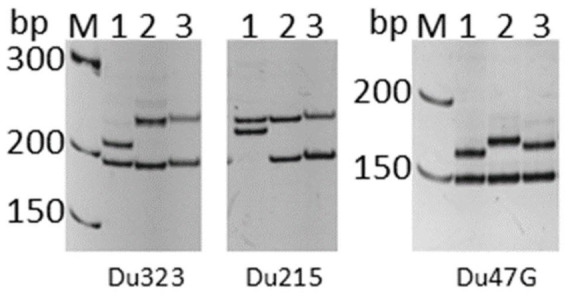
Electrophoretic fractionation of PCR amplification products of microsatellite loci Du323, Du215, and Du47G, where M—Low range DNA ladder (Thermo Scientific, Waltham, MA, USA): 1—*D. unisexualis* (Jradzor, Armenia); 2—putative *D. uzzelli* (Jradzor, Armenia); 3—*D. uzzelli* (ZISP-21212-2).

Microsatellite analyses revealed clear differences between *D. uzzelli* and *D. unisexualis* in fragment size, microsatellite cluster structure, and point mutations in the flanking regions (Figure 4). At locus Du323, significant differences in fragment size were due to variation in the number of repeat copies, while alleles of the same size showed no sequence differences. The allele structures at loci Du323 and Du215 in *D. uzzelli* (ZISP-21212-2) matched those of the Jradzor specimens and were clearly distinct from *D. unisexualis.* At the Du47G locus, alleles from the Jradzor and Kars specimens differed by a single tetranucleotide repeat (Figure 4).

## 4. Discussion

Parthenogenesis in vertebrates was first discovered among reptiles in the genus *Darevskia*, establishing that all-female populations could exist through natural obligate unisexual reproduction over long evolutionary periods [1,2,10]. Reticulate evolution serves as one of the drivers of diversity in this genus, where new lineages emerge through the merging of ancestral lines via interspecific hybridization [9,10,11]. Consequently, the parthenogenetic species *D. uzzelli* arose through interspecific hybridization between “paternal” species *D. valentini* and “maternal” species *D. raddei* [8,9,10]. Similar to other parthenogenetic species, *D. uzzelli* clonal reproduction represents an important model for investigating the mechanisms of hybrid speciation and the long-term maintenance of parthenogenesis in vertebrates [1,10].

The discovery of *D. uzzelli* in Armenia validates a long-standing biogeographical hypothesis proposed by Darevsky and Danielyan [4], who predicted the potential occurrence of this species in the region based on the continuity of mountain steppe and river gorge habitats extending from the Kars Plateau in Türkiye into Shirak Province in Armenia. Our finding in the Akhuryan River valley, approximately 80 km from the nearest known Turkish locality in Kars, confirms that the river acts as a corridor rather than a barrier for this species. It is therefore likely that the species’ distribution is more continuous across this landscape than previously understood, with populations potentially connecting the Armenian locality with those in the Araks and Murat River valleys in Türkiye [30].

The new locality is situated near Jradzor village in Shirak Province, northwestern Armenia, on the right bank of the Hoghmajur River, a tributary of the Akhuryan River in the Armenian Highlands. The surrounding area is characterized by dry, open river valleys, rocky outcrops, and steppe grasslands that provide suitable microhabitats for rock lizards. These habitats typically include abundant sun-exposed stones on slopes. Vegetation is sparse to moderately grassy, with scattered shrubs and low herbaceous cover, forming typical microhabitats that support the daily and seasonal activity cycles of rock lizards. In Türkiye, *D. uzzelli* is similarly associated with stony substrates that provide shelter, combined with sparsely distributed herbaceous vegetation.

The newly discovered population in Jradzor is unique, as it represents the largest known assemblage of parthenogenetic species at a single site worldwide, where four species live together (Figure 1). This population also reveals the gap in the currently recognized biogeographic distributions of *D. unisexualis*, *D. armeniaca*, and *D. dahli* species. Although all four parthenogenetic species were recorded together at the Jradzor locality, this area lies outside the currently recognized distribution ranges of these taxa. The newly discovered sympatric assemblage therefore not only extends the known range of *D. uzzelli*, but also highlights the need for updated regional surveys and revision of current biogeographic assessments. Typically, *D. uzzelli* coexists with its most closely related species, *D. unisexualis*, due to their similar microhabitat preferences [1,4,8]. Two of them, *D. armeniaca* and especially *D. dahli*, tend to inhabit forested areas, reflecting the humid habitat preferences of their paternal species, *D. mixta* [1,4,8]. A known three-species assemblage (*D. armeniaca, D. dahli,* and *D. rostombekowi*) coexists near Dilijan city along the river gorge close to forests, which matches their ecological preferences [1]. In contrast, the newly discovered four-species composition in Jradzor includes areas not typically preferred by *D. dahli*, but it maintains the highest proportion there (Figure 1). Based on our expert assessment, *D. unisexualis* is the next most abundant species at this site, followed by *D. armeniaca* and *D. uzzelli*.

Pholidotic and genetic data indicate that the Armenian *D. uzzelli* specimens fall well within the documented range of variation observed in Turkish populations and correspond more closely to the holotype collected by R. and E. Clark in 1967 (CAS 105689; Figure S1) and associated paratypes (Figure 3 and Figure S1) than to populations from the vicinity of Horasan. The mitochondrial DNA profile of the Jradzor specimen matches that of *D. raddei* from Kars, which is also the established maternal ancestor of *D. uzzelli* [9,27].

Our results clearly distinguish *D. uzzelli* morphologically from the closely related parthenogenetic species *D. unisexualis* [9], with which it often coexists in Türkiye [30]. Three key features can be used to differentiate these species: *D. uzzelli* has a greenish-brown dorsal ground color, enlarged dorsal scales along the ventral plates, and a rostral scale that does not contact the frontonasal scale. Because *D. uzzelli* is sympatric with *D. dahli* in the Jradzor population, the latter can be distinguished by its lighter yellow ventral coloration and a dorsal pattern of well-defined, separate dark blotches that do not merge. *D. armeniaca* is the most genetically distant species from *D. uzzelli* and exhibits distinct morphological traits [14].

Our genetic analyses confirmed the identity of the specimens as *D. uzzelli*, providing strong support for the morphological identification. One of the key findings of our intraspecific analysis of different populations of *D. uzzelli* is the high degree of morphological variation within the species. Comparison of pholidosis between the Armenian and Turkish populations confirms their taxonomic identity while simultaneously revealing considerable diversity both among populations and within each population of *D. uzzelli*. This result is particularly important in light of the complex taxonomic history of closely related species from Türkiye. *D. uzzelli* was described in 1977 [4], and later *D. sapphirina* was recognized as an independent species within this group [5]. Currently, *D. sapphirina* is considered conspecific with *D. bendimahiensis* [2,11]. DNA barcoding has confirmed close relationships between *D. uzzelli* and *D. unisexualis*, as well as between *D. sapphirina* and *D. bendimahiensis* [31].

In this context, the significant morphological variability documented in *D. uzzelli* suggests substantial clonal diversity, which is of particular evolutionary interest in parthenogenetic species. Some variability has been observed in other parthenogenetic species of *Darevskia*, where morphological diversity reflects underlying clonal variation. The minor allelic variability detected at the Du47G microsatellite locus, represented by a single difference in tetranucleotide repeats, is best interpreted as intraspecific polymorphism, likely resulting from the accumulation of mutations in geographically separated populations. Overall, this level of morphological and genetic variability helps to clarify the taxonomic characterization of the poorly studied *D. uzzelli* and may also contribute to resolving broader questions related to limited genetic and phenotypic heterogeneity in parthenogenetic reptiles. Further research along the Akhuryan River valley and adjacent areas may help to refine its distribution range, estimate population size, and develop effective conservation strategies for this newly recorded species in Armenia.

The Jradzor area in Armenia is of exceptional scientific and ecological importance as the only known site hosting the highest concentration of parthenogenetic lizards globally. This population is a critical refuge for rare endemics with highly limited distributions, including also *D. dahli* and *D. unisexualis* [32,33]. Notably, this site is the single location for *D. uzzelli* in Armenia, which, due to its extreme rarity and restricted range, carries a Critically Endangered (CR) status for inclusion in the new edition of the Red Book of Armenia. These findings require immediate management, as most habitats for these endangered lizards are currently located outside of protected areas and are under intense pressure from human activity. The preservation of this site is particularly urgent because it is currently undergoing the construction of a Kaps Reservoir, a process that causes direct habitat loss and irreversible changes to the microclimate necessary for the lizards’ survival. Protecting this unique place of coexistence is essential for conserving Armenia’s natural heritage.

## 5. Conclusions

Our study supports the hypothesis that the parthenogenetic species *Darevskia uzzelli* is not strictly endemic to Türkiye and occurs across the river border in Armenia in a similar landscape. This finding extends the known distribution range of the species by approximately 80 km to the northeast. Comparison populations by external morphology and microsatellites reveal that *D. uzzelli* is a highly diverse group that is unusual for a clonal species. Moreover, Jradzor locality in Armenia is unique as the only known site where four parthenogenetic species (*D. uzzelli*, *D. armeniaca*, *D. dahli*, and *D. unisexualis*) coexist in sympatry. The presence of four clonal lineages at a single site makes this population a critical “natural laboratory”. This high diversity of parthenogenetic species provides a rare opportunity to study ecological niche divergence, competitive exclusion, and the evolutionary ecology of unisexual lizards.

*Darevskia uzzelli* is classified as Endangered on the IUCN Red List due to its restricted range in Türkiye, and its discovery in Armenia has important conservation implications. We therefore recommend including *D. uzzelli* in the Red Data Book of Armenia.

## Figures and Tables

**Table 1:** Diagnostic morphological characters in three populations of *Darevskia uzzelli*.

Character	Jradzor (*n* = 2)	Horasan (*n* = 20)	Erzrum (*n* = 6)
MBSN	45, 45	53.9 (51–56)	51.0 (48–54)
VSN	24, 25	23.4 (22–24)	24.7 (24–25)
CSN	12, 12	11.6 (10–13)	11.8 (10–12)
GSN	27, 27	29.3 (26–35)	26.2 (25–28)
FPN	18, 18	19.7 (18–21)	18.7 (17–21)
SDLN	23, 24	25.1 (23–27)	23.9 (22–25)
SCGN	12, 14	11.2 (9–12)	11.4 (9–13)
SMN	1, 1	1.1 (1–3)	1.8 (1–3)
MTN	2, 1	2.5 (2–3)	2.8 (2–3)
PTMN	2, 2	2.8 (2–4)	2.5 (2–3)

## Data Availability

All data generated or analysed during this study are included in the Supplementary Materials. Further inquiries can be directed to the corresponding authors.

## Supplementary Materials

The following supporting information can be downloaded at: https://www.mdpi.com/article/10.3390/ani16142140/s1, Figure S1: Holotype of *D. uzzelli* CAS-105689 lizard. Type locality: 25 km S from Kars, Türkiye. Photo by D.A. Melnikov, Table S1: Raw data of the morphology of *D. uzzelli*.
