# Supplementary figures and images for: The Unique Sympatric Population of Uzzell’s Lizard (Darevskia uzzelli, Lacertidae, Squamata) Reveals Clonal Diversity and Urgent Conservation Value
Source: Animals (Basel). 2026 Jul 9;16(14):2140. doi: 10.3390/ani16142140 (PMC13403777; doi:10.3390/ani16142140)

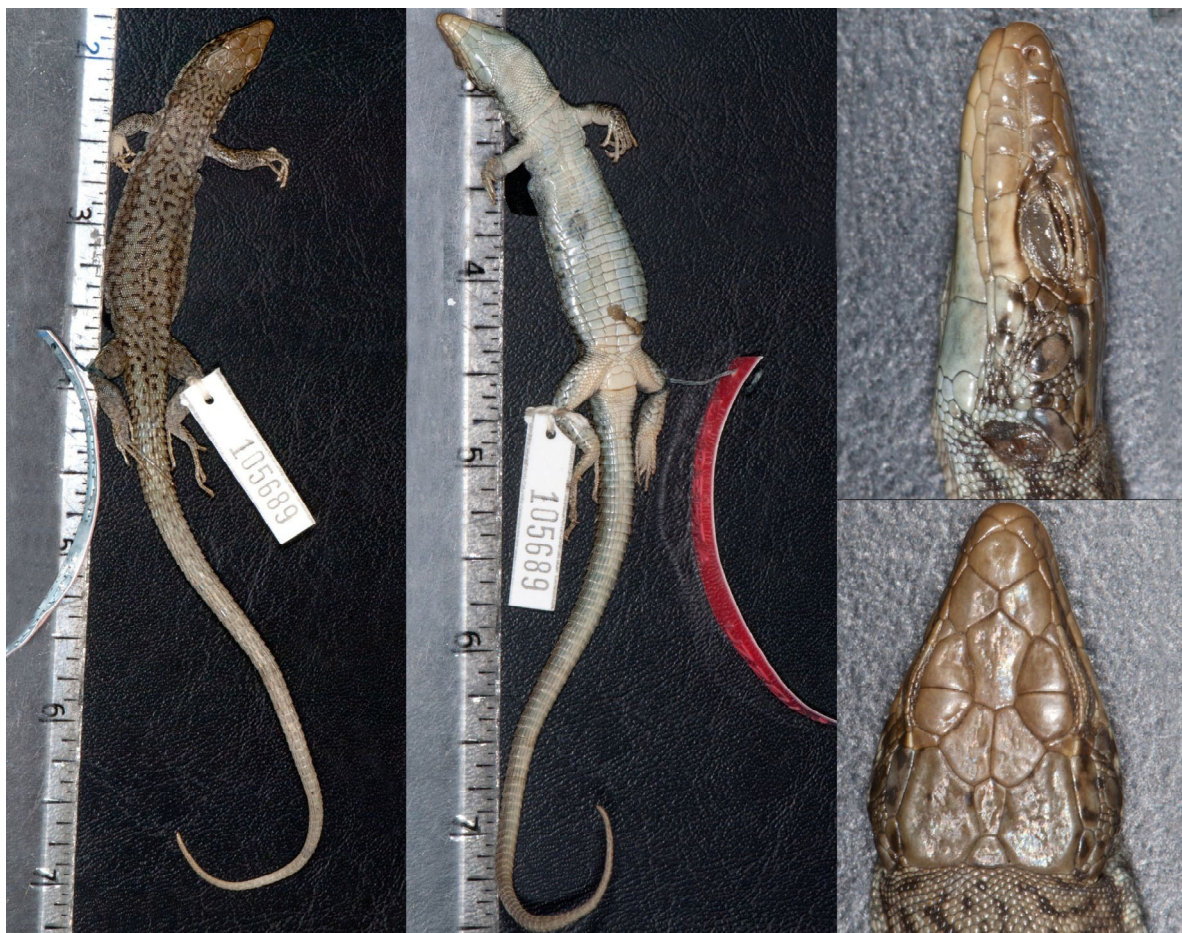

Supplement: Supplementary file 1 [file animals-16-02140-s001.zip › Figure S1.pdf]
